# Green Synthesis of Silver Nanoparticle Using Black Mulberry and Characterization, Phytochemical, and Bioactivity

**DOI:** 10.3390/antibiotics13080686

**Published:** 2024-07-24

**Authors:** Yoo-Na Jeon, Su-Ji Ryu, Ha-Yeon Lee, Jang-Oh Kim, Jong-Suep Baek

**Affiliations:** 1 Department of Bio-Health Convergence, Kangwon National University, Chuncheon 24341, Republic of Korea; 2 Department of Radiological Science, Kangwon National University, Samcheok 25949, Republic of Korea; 3 Department of Bio-Functional Materia, Kangwon National University, Samcheok 25949, Republic of Korea; 4 BeNatureBioLab, Chuncheon 24206, Republic of Korea

**Keywords:** silver nanoparticles, black mulberry, antioxidant, antibacterial, anti-inflammation, anticancer

## Abstract

Synthesis of silver nanoparticles (AgNPs) using plant extracts has been proposed as a more advantageous and environmentally friendly alternative compared to existing physical/chemical methods. In this study, AgNPs were synthesized from silver nitrate using black mulberry (BM) extract. The biosynthesized AgNPs were characterized through an UV-visible spectrometer, X-ray diffraction, and transmission electron microscopy. Additionally, BM-AgNPs were subjected to antioxidant, antibacterial, anti-inflammatory, and anticancer activities. AgNPs biosynthesized from BM extract were dark brown in color and showed a strong peak at 437 nm, confirming that AgNPs were successfully synthesized. The size of AgNPs was 170.17 ± 12.65 nm, the polydispersity index was 0.281 ± 0.07, and the zeta potential value was −56.6 ± 0.56 mV, indicating that the particles were stable. The higher total phenol, flavonoid, and anthocyanin content of BM-AgNPs compared to BM extract indicates that the particles contain multiple active substances due to the formation of AgNPs. The DPPH and ABTS assays showed decreased IC_50_ values compared to BM extract, demonstrating improved antioxidant activity. AgNPs inhibited the growth of *S. aureus* and *E. coli* at 600 μg/mL, with minimum bactericidal concentrations determined to be 1000 and 1200 μg/mL, respectively. The anti-inflammatory activity was 64.28% at a BM-AgNPs concentration of 250 μg/mL. As the concentration increased, the difference from the standard decreased, indicating the inhibitory effect of AgNPs on bovine serum albumin denaturation. The viability of MCF-7 cells treated with BM-AgNPs was found to be significantly lower than that of cells treated with BM extract. The IC_50_ value of BM-AgNPs was determined to be 96.9 μg/mL. This study showed that BM-AgNPs have the potential to be used in the pharmaceutical industry as antioxidant, antibacterial, anti-inflammatory, and anticancer agents.

## 1. Introduction

The field of nanotechnology research has made significant progress over the past few decades and has emerged as a promising technology in the scientific field, particularly in the development of materials with potential medical applications [1]. Metal nanoparticles, including gold, silver, copper, platinum, iridium, osmium, and palladium are being increasingly used in biomedical applications due to their biological activity [2,3].

Silver (Ag) is the metal most often used in nanoparticle preparation due to its antifungal, antibacterial, antiviral, anticancer, and antioxidant properties [4,5]. Silver nanoparticles (AgNPs) have been used as an antibacterial agent due to their effectiveness in sterilizing the growth inhibition of various microorganisms, such as *Vibrio cholerae*, *Candida albicans*, *Pseudomonas aeruginosa*, *Escherichia coli* (*E. coli*), and *Staphylococcus aureus* (*S. aureus*) [6]. AgNPs have been reported to induce selective death of cancer cells, and can play a role in drug delivery and act as a promising therapeutic agent against cancer [7]. AgNPs are synthesized using organisms such as algae, plants, bacteria, fungi, and their metabolites, which act as reducing agents, stabilizers, and capping agents [8]. A synthetic method using such biological materials is cost-effective, efficient, and environmentally friendly [9]. AgNPs prepared using biological materials have the properties of a high surface area, small size, and high dispersion [10]. Among them, plants are considered the preferred source because they are highly productive and easily available [11]. Plants such as leaves, fruits, and seeds used in green synthesis contain a variety of natural phytochemicals such as terpenoids, flavonoids, alkaloids, phenols, saponins, tannins, and amino acids [12,13,14]. By these phytochemicals, Ag ions are stabilized and reduced to nanoparticles, and improve the biological activity of nanomaterials [15]. The method of synthesis with plant extracts is effective in preventing oxidation and aggregation of synthesized AgNPs while reducing cost and chemical usage [16].

Mulberry, belonging to the genus Morus, has been distributed worldwide in diverse climatic conditions from temperate to tropical [17]. The three types of mulberry fruit are red mulberry (*Morus rubra* L.), black mulberry (*Morus nigra* L.) (BM), and white mulberry (*Morus alba* L.) [18]. Among them, BM belongs to the Moraceae family and is a purple to black fruit with a distinctive, slightly acidic taste [19]. BM is a fruit traditionally known for its use in natural medicine because of its nutritional properties and taste as well as its high content of various phytochemicals, including flavonoids and phenolic compounds, especially anthocyanins [20]. The major anthocyanins identified in BM are cyanidine-3-glucoside and cyanidine-3-lutinoside (C3G) [21], and the C3G is considered the most commonly occurring and important molecule among anthocyanins due to its diverse biological activities [22]. BM has been reported for various biological activities including antioxidant, anticancer, anti-inflammatory, antibacterial, neuroprotective, hyperlipidemia, antiatherosclerosis, and anti-obesity agents due to the bioactive content of polyphenols and anthocyanins [23,24,25].

This study aimed to synthesize AgNPs from BM extracts and evaluate their various biological activities. The anti-inflammatory and anticancer activities of BM-AgNPs are reported here for the first time. A UV-visible spectrometer (UV-vis spectra), dynamic light scattering (DLS), X-ray diffraction (XRD), transmission electron microscopy (TEM) and energy dispersive X-ray spectroscopy (EDS) were performed to characterize the size, shape, dispersion, crystallinity, stability, and surface morphology of the biosynthesized nanoparticles. Through the total phenolic content (TPC) and total flavonoid contents (TFC) of BM-AgNPs, the content of phytochemicals such as polyphenols and flavonoids contained in BM-AgNPs was measured, and the C3G content was measured through TAC. In addition, 2,2-diphenyl-1-picrylhydrazide (DPPH) and ‘2,2’-azino-bis (3-ethylbenzothiazoline-6-sulfonic acid) (ABTS) experiments were performed to confirm the antioxidant capacity of BM-AgNPs. The antibacterial activity of the prepared BM-AgNPs was analyzed on Gram-positive (*E. coli*) and Gram-negative (*S. aureus*). The anti-inflammatory effect of BM-AgNPs was evaluated through protein denaturation experiments using bovine serum almumin (BSA), while the anti-cancer properties of BM-AgNPs were investigated using a breast cancer (MCF-7) cell line.

## 2. Results and Discussion

### 2.1. Characterization of BM-AgNPs

#### 2.1.1. UV-Vis Absorbance Spectra and Color Change

**Figure 1:**
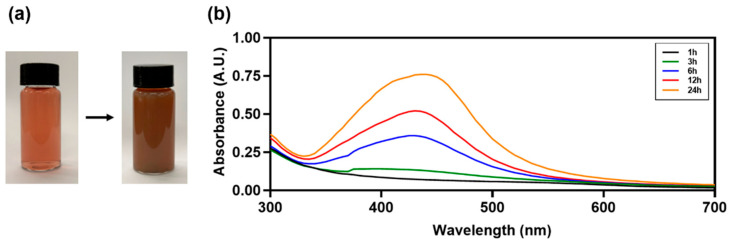
(**a**) Image of Ag colloids synthesized with BM extract after 24 h, (**b**) UV-Vis spectra of BM-AgNPs synthesized from different incubation times (1, 3, 6, 12, and 24 h).

The color change observed in AgNPs confirms the reduction of Ag ions in silver nitrate (AgNO_3_) [26]. The reaction mixture containing AgNO_3_ and BM extract turned dark brown, confirming the formation of BM-AgNPs (Figure 1a). UV-vis spectral analysis is used to obtain information about AgNPs, such as their stability with regard to size and shape, in aqueous suspensions [27]. In the absorption spectrum of AgNPs, the surface plasmon resonance band appears in a broad range of 300–700 nm, with a strong absorption band observed at 437 nm (Figure 1b). This absorption spectrum broadened with an increasing absorption peak up to 24 h. This is due to the distinct size distribution of BM-AgNPs and indicates the presence of polydisperse AgNPs in the solution [28,29]. The polydisperse presence of AgNPs in the solution may be due to the slow reduction rate [30].

#### 2.1.2. TEM

**Figure 2:**
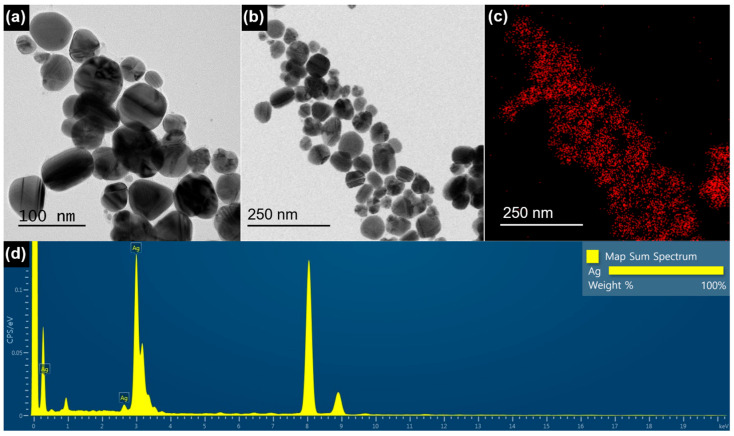
(**a**,**b**) TEM images of BM-AgNPs with different magnification, (**c**) EDS mapping of Ag, and (**d**) EDS spectrum of BM-AgNPs.

TEM images provide information about the size, morphology, and shape of the biosynthesized BM-AgNPs. Through TEM analysis of BM-AgNPs, it was confirmed that the particles were spherical below 100 nm (Figure 2a,b). Spherical particles have a large surface area to prevent aggregation and are relatively evenly distributed [31]. In Figure 2c, EDS mapping confirmed the presence of Ag ions in the AgNPs. In the EDS spectrum, Ag was identified by a strong and distinct peak at approximately 3 keV (Figure 2d). This finding is consistent with a previous study, which also confirmed that AgNPs exhibit a strong peak at 3 keV, aligning with the EDS spectrum results of BM-AgNPs [32].

#### 2.1.3. Particle Size, PDI and Zeta Potential

The particle size, PDI, and zeta potential of BM-AgNPs were measured (Table 1). DLS was performed to measure the size distribution of AgNPs, and the particle size of BM-AgNPs was measured to be 170.17 ± 12.65 nm. DLS is an indirect ensemble technique based on the luminosity signal of particles, which can bias particle size measurements toward larger sizes [33,34]. Therefore, diameters obtained through DLS are generally larger than those measured through TEM [35]. PDI values less than 0.1 are considered monodisperse [36]. The PDI value of BM-AgNPs is 0.281 ± 0.07, which is considered polydisperse, and the broad absorption peak in Figure 1 mentioned above supports the existence of polydisperse AgNPs in the solution. BM-AgNPs were measured with a PDI of 0.281 ± 0.07, which is relatively monodisperse. The determination of the zeta potential serves as a key factor in assessing the stability of AgNPs. Zeta potential is an indicator of the magnitude of electrostatic interactions between dispersed particles and can indirectly evaluate the stability of nanoparticles in colloidal suspensions by reflecting their ability to electrostatically repel each other [37,38]. Particles with zeta potential values exceeding +30 mV or dropping below −30 mV are considered stable [39]. The zeta potential value of BM-AgNPs was −56.6 ± 0.56 mV, confirming that the particles were in a stable condition. Therefore, BM-AgNPs can prevent aggregation of the particles and maintain stability in the solution due to the electrostatic repulsion between them.

#### 2.1.4. XRD

**Figure 3:**
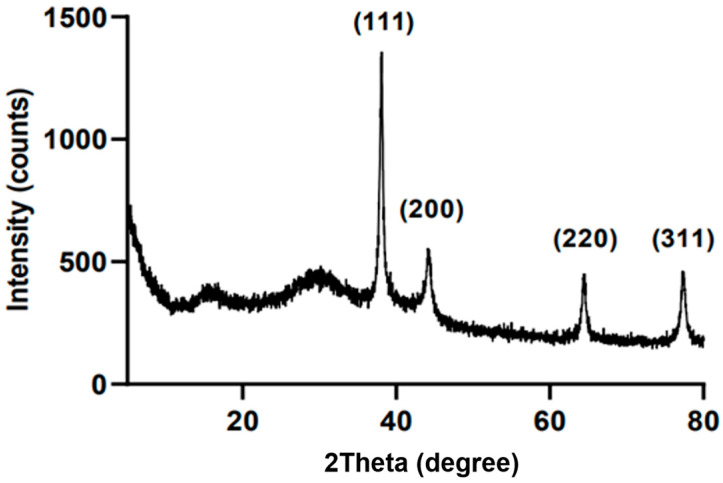
XRD pattern of BM-AgNPs.

The crystalline nature of BM-AgNPs was confirmed through analysis of the XRD pattern (Figure 3). The XRD analysis revealed the presence of four prominent diffraction peaks at specific 2θ values: 38.12° (111), 44.12° (200), 64.40° (220), and 77.26° (311). The high intensity diffraction peak at (111) shows the growth direction of the nanocrystals [40]. It was confirmed that these peaks are due to the crystallographic plane of the BM-AgNPs by face-centered cubic (fcc) information provided in JCPDS (Joint Committee Powder Diffraction Standards) [41].

### 2.2. Phytochemical of BM-AgNPs

Table 2 shows the TPC, TFC, TAC, and antioxidant results of BM extract and BM-AgNPs. The TPC of BM extract was 24.57 ± 0.17 mg/g, BM-AgNPs was 148.42 ± 2.33 mg/g, showing the higher phenol content of BM-AgNPs. The TFC was 8.07 ± 0.04 mg/g for BM extract and 12.80 ± 0.65 mg/g for BM-AgNPs, confirming that BM-AgNPs had a higher flavonoid content. Anthocyanins are a group of water-soluble flavonoids that relieve oxidant stress responses by scavenging free radicals [42,43]. As a result of the TAC, it was confirmed that the C3G content of BM-AgNPs was higher at 1.04 ± 0.24 mg/L for the BM extract and 1.63 ± 0.60 mg/L for BM-AgNPs, which showed that anthocyanin was used to obtain metal nanoparticles in the process. Previous studies have reported the reduction of metal ions and stabilization of AgNPs by polyphenols and anthocyanins present in blackcurrant extract [44].

**Figure 4:**
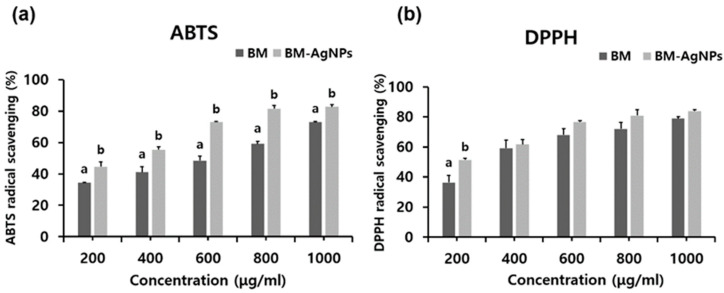
Antioxidant activities of BM extract and BM-AgNPs. (**a**) DPPH radical scavenging activity, (**b**) ABTS radical scavenging activity. Data are expressed as means ± standard deviation (n = 3). Different letters (a, b) in the same concentrations (*p* < 0.05) indicate significant differences.

In ABTS, IC_50_ values were 575.48 ± 21.69 μg/mL for the BM extract and 257.09 ± 35.44 μg/mL for BM-AgNPs. In DPPH, IC_50_ values were 334.63 ± 23.98 μg/mL for the BM extract and 100.94 ± 15.01 μg/mL for BM-AgNPs. It was found that BM-AgNPs have a stronger effect on neutralizing free radicals. The DPPH and ABTS radical scavenging of BM extracts and BM-AgNPs are shown in Figure 4. At all concentrations, the ABTS radical scavenging value increased in a concentration-dependent manner and was higher for BM-AgNPs than for BM extracts. The DPPH radical scavenging value also increased in a concentration-dependent manner at all concentrations and was higher for BM-AgNPs than for BM extracts. Due to the different radical scavenging mechanisms of DPPH and ABTS, significant differences between extracts and AgNPs in DPPH radical scavenging were only identified at 200 μg/mL [45]. The higher TFC, TPC, and TAC of BM-AgNPs in comparison to the BM extract may be associated with an enhanced antioxidant activity. This indicates that the active molecules present in the extract utilized as a reducing agent may elevate the antioxidant activity of BM-AgNPs [46].

### 2.3. Antibacterial Activities

The results of minimum inhibitory concentration (MIC) and minimum bactericidal concentration (MBC) on the BM extract and BM-AgNPs at various concentrations are shown in Table 3. In the MIC assay, the lowest antimicrobial concentration preventing growth is shown, while the MBC assay is determined by the minimal concentration causing microbial death [47]. A specific MIC indicating inhibition implies the possibility of organism proliferation, unlike MBC, which causes death [48]. Antibacterial effects against *S. aureus* and *E. coli* were observed using the BM extract and BM-AgNPs. The BM extract did not show antibacterial activity up to 1000 μg/mL, and the MIC of BM-AgNPs were 600 μg/mL in *S. aureus* and 600 μg/mL in *E. coli*. The MBC for BM-AgNPs were 1000 μg/mL in *S. aureus* and 1200 μg/mL in *E. coli*. These results are attributed to differences in the thickness, structure, and composition of bacterial cell walls and membranes [49]. The cell wall of Gram-negative bacteria is thinner and more sensitive to AgNPs than positive bacteria [50], while the thick peptidoglycan layer of Gram-positive bacteria constitutes a solid barrier that makes it difficult for AgNPs to adhere and penetrate [51]. AgNPs have a small particle size and a large surface area that can contact bacteria and easily cause cell membrane damage when silver ions penetrate bacterial cells through cell wall destruction [52,53]. Although antibacterial activity was not confirmed in the BM extract, BM-AgNPs showed a notable antibacterial effect. The results obtained were consistent with previous studies that found that nanoparticles synthesized using plant extracts, such as mulberry leaf [54], *Curcuma longa* [55], and *Carduus crispus* [56], exhibited high antibacterial properties.

### 2.4. Anti-Inflammatory

**Figure 5:**
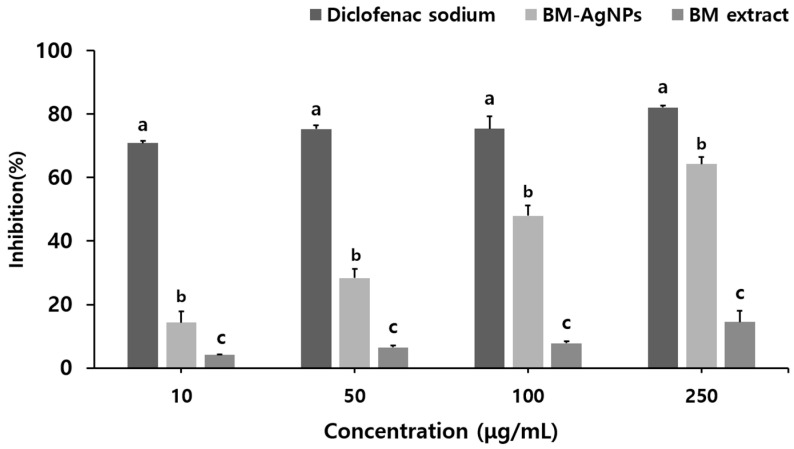
Anti-inflammatory activity of BM extract and BM-AgNPs. Data are expressed as means ± standard deviation (n = 3). Different letters (a–c) in the same concentrations (*p* < 0.05) indicate significant differences.

In this study, BSA assay was used to investigate the anti-inflammatory activity of the BM extract and BM-AgNPs at different concentrations of 10, 50, 100, and 250 μg/mL. Inflammation is caused by the denaturation of proteins, a process in which the secondary and tertiary structures of proteins are damaged by stress compounds such as strong acids, bases, and heat [57]. BSA accounts for 60% of the total protein in animal serum, and when heated, it denatures into protein and begins to express antigens associated with type 3 hypersensitivity reactions associated with diseases such as rheumatoid arthritis and serum sickness [58]. The anti-inflammatory effect of BM-AgNPs was shown to inhibit BSA protein denaturation to a higher extent than that of the BM extract, with the level of inhibition increasing with the increasing concentration (Figure 5). The BM extract showed an albumin denaturation inhibition effect of 4.21, 6.52, 7.81, and 14.52% at each concentration, and BM-AgNPs showed an albumin denaturation inhibition effect of 14.29, 28.37, 47.90, and 64.28%. The standard, diclofenac sodium showed an inhibitory effect of 70.84, 75.26, 75.32, and 82.09%. It was confirmed that the difference in inhibitory activity between the BM extract and BM-AgNPs increased as the concentration increased. The inhibitory activity of BM-AgNPs became similar to that of Diclofenac sodium as the concentration increased. These results suggest that AgNPs may exhibit anti-inflammatory properties similar to diclofenac sodium at concentrations above 250 μg/mL. These results affirmed the regulation of autoantigen production responsible for protein denaturation in BM-AgNPs and demonstrated the inhibitory effect of AgNPs on BSA denaturation.

### 2.5. Anti-Cancer

**Figure 6:**
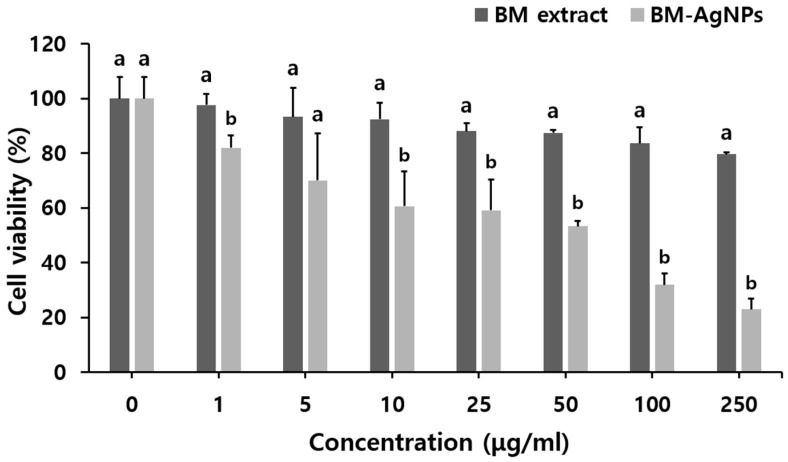
Anti-cancer activity of BM extract and BM-AgNPs. Data are expressed as means ± standard deviation (n = 3). Different letters (a, b) in the same concentrations (*p* < 0.05) indicate significant differences.

The *in-vitro* cytotoxic activity of the BM extract and BM-AgNPs against MCF-7 cells was evaluated using the MTT assay (Figure 6). The BM extract and BM-AgNPs exhibited cytotoxic effects in a dose-dependent manner, with cell viability significantly decreasing with increasing concentration of AgNPs tested compared to the extract. In the BM extract, cytotoxicity was 79.70% at 250 μg/mL, while BM-AgNPs showed a high cytotoxic effect of 22.99%. The concentration that can reduce the cell viability of MCF-7 cells by 50% is the IC_50_, which is 96.9 μg/mL for BM-AgNPs. AgNPs were shown to stimulate and induce reactive oxygen species (ROS) by inhibiting the synthesis of intracellular antioxidant systems [59]. This damage extends to cellular components such as cell membranes and DNA, resulting in cell death [60].

## 3. Materials and Methods

### 3.1. Materials

Potassium acetate was obtained from Junsei Chemical (Tokyo, Japan). Folin-Ciocalteu’s phenol reagent (F9252), AgNO_3_, and sodium carbonate were purchased from Daejung Chemical (Siheung, Republic of Korea). Phosphate-buffered saline (PBS) was used without a further purification system. Gallic acid (G7384), quercetin (Q4951), and bovine serum albumin (A2153) were obtained from Sigma-Aldrich (St. Louis, MO, USA). Diclofenac sodium salt (D2508) was purchased from TCI (Tokyo, Japan). Mueller hinton broth (MHB) and agar were bought from Kisanbio (Seoul, Republic of Korea). Dimethyl sulfoxide (DMSO) for cell culture was purchased from Gen DEPOT (Altair, TX, USA). Dulbecco’s Modification of Eagle’s Medium (DMEM), penicillin-streptomycin, and fetal bovine serum (FBS) were purchased from Thermo Fisher Scientific (Waltham, MA, USA). 

### 3.2. Preparation of BM Extract

BM powder was obtained by purchasing fresh mulberry fruit from a farmer in Wanju, Republic of Korea, and freeze-drying it at −50 °C. 1 g of BM powder was placed into 50 mL of distilled water (DW), followed by ultrasonic extraction at 40 °C for 30 min. The extract was filtrated through Whatman 6 filter paper at room temperature. The aqueous extract was stored at 4 °C, intended for later applications.

### 3.3. Synthesis of BM-AgNPs

The solution of AgNO_3_ at a concentration of 3 mM was mixed with the BM extract in a ratio of 9:1 (AgNO_3_: BM extract). The mixture was stored at 60 °C for 24 h. Afterward, the mixture was centrifuged at 13,000 rpm for 20 min. DW was then added, and the pellet was washed three times to eliminate any remaining residues of Ag ions and extract.

### 3.4. Characterization of BM-AgNPs

The analysis of AgNPs synthesis involved the use of a UV-Vis spectrometer (BioMate 160, Thermo Fisher Scientific, Waltham, MA, USA) at 300–700 nm, employing a cuvette cell containing 1 mL of the sample. The particle size, polydispersity index (PDI), and zeta potential were measured in a Mastersizer 2000 (Malvern Instruments, Malvern, UK). Morphological details were observed by loading AgNPs samples onto copper grids and employing TEM with EDS capabilities (JEM-2100F, JEOL, Tokyo, Japan). The crystalline structure of AgNPs was analyzed using XRD patterns over 2θ angles ranging from 5 to 80°, employing an X-ray diffractometer (MPD, PAN Analytical, Almelo, The Netherlands).

### 3.5. Phytochemical of BM-AgNPs

#### 3.5.1. TFC

A modified aluminum chloride colorimetric method was used to measure the TFC of BM-AgNPs [61]. 400 μL of each sample was added along with 20 μL of 10% aluminum chloride and 20 μL of 1 M potassium acetate. 1 mL of DW was added to the mixture and reacted for 30 min. The mixture’s absorbance was evaluated at a wavelength of 415 nm. For calibration, a range of quercetin standard solutions (31.25, 62.5, 125, 250, and 500 μg/mL) was utilized.

#### 3.5.2. TPC

Quantification of the TPC in BM-AgNPs was performed using the Folin-Ciocalteu colorimetric method, which operates according to the oxidation−reduction principle, with slight modifications [62]. A total of 0.2 mL of the sample and 0.6 mL of the DW was mixed in a test tube. A total of 1 mL Folin-Ciocalteu reagent 0.8 mL and 7.5% sodium carbonate solution of were mixed and added to the test tube. Room temperature incubation was maintained for 45 min and the mixture’s absorbance was evaluated at a wavelength of 760 nm. The standard used was a gallic acid solution.

#### 3.5.3. TAC

TAC quantification of BM-AgNPs was performed using the pH difference method with slight modifications [63]. A potassium chloride buffer (pH 1.0) was prepared by combining a 0.2 M hydrochloric acid (HCl) solution with a 0.2 N potassium chloride (KCl) solution. A sodium acetate buffer (pH 4.5) was created using a 0.4 M sodium acetate solution, with pH adjustment achieved through the addition of acetic acid. The sample (1 mg/mL) was diluted ten-fold with a buffer solution. The absorbance was evaluated at 510 to 700 nm. The calculation of the sample’s TAC (mg of C3G/L) was performed utilizing the relevant Equation (1):

TAC (mg/L) = A × M × DF × 1000/(ε × d)(1)

A (absorbance) = (A _510nm_ − A _700nm_) _pH = 1.0_ − (A _500nm_ − A _700 nm_) _pH = 4.5_
M (molecular weight) = 449.2 g/mol for C3G
DF (dilution factor)
1000 = conversion from g to mg
ε (molar absorptivity coefficient in L/mol/cm for C3G) = 26,900
d (path length) = 1 cm

### 3.6. Antioxidant Activity

#### 3.6.1. ABTS

The scavenging activity of ABTS radicals was measured based on published methods [64]. Potassium persulfate and ABTS^+^ were adjusted to 2.45 mM and 7 mM, respectively, and then mixed in a 1:1 ratio. Darkness was maintained for 16 h during the reaction of the mixture. The solution was suitably diluted using 0.1 M PBS (pH 7.4) until an absorbance of 0.9 at 730 nm was achieved. The BM extract and BM-AgNPs were diluted to various concentrations (200, 400, 600, 800, and 1000 μg/mL). A mixture consisting of 30 μL of the extract and 150 μL of the ABTS solution was prepared and reacted for 25 min in a dark environment. The absorbance was measured at 730 nm. Ascorbic acid solutions were employed for calibration purposes.

#### 3.6.2. DPPH

The scavenging activity of DPPH radicals was measured with a slightly modified method [65]. To measure the scavenging activity of DPPH radicals, the BM extract and BM-AgNPs were diluted to various concentrations (200, 400, 600, 800, and 1000 μg/mL) and then mixed with 0.4 mM DPPH solution in a 1:1 ratio. The mixture was incubated in a dark condition (room temperature, 20 min) and the mixture’s absorbance was evaluated at a wavelength of 517 nm. Ascorbic acid was used as a standard and the concentrations were 20, 40, 60, 80, and 100 μg/mL.

### 3.7. Antibacterial Activity

The strain of *E. coli* and *S. aureus* was used for the test. The strain of *E. coli* and *S. aureus* was grown in MHB and incubated overnight at 37 °C. Each sample was diluted to an appropriate concentration. DW was used as a negative control. Bacteria were diluted with MHB until 0.5 at 600 nm. 100 μL of the sample and bacteria were treated in a 96-well plate, respectively. The absorbance was evaluated at a wavelength of 600 nm at 0 and 24 h. The experiment was conducted three times. The bactericidal effect of the biosynthesized AgNPs was determined through a solid medium (Mueller Hinton Agar (MHA)) culture. Dispense 50 μL of the sample onto MHA and incubate at 37 °C for 24 h to calculate the colony forming unit (CFU) value.

### 3.8. Anti-Inflammatory

The anti-inflammatory test of BM-AgNPs was measured using bovine serum albumin (BSA) with some modifications [66]. A total of 0.45 mL of 1% BSA was mixed with 0.5 mL of BM extract and BM-AgNPs of various concentrations. The reaction was incubated at 37 °C for 20 min, then the temperature was gradually increased to 60 °C and heated for 20 min. Diclofenac sodium at different concentrations of 0.5 mL was used as a positive control. Protein inhibition by AgNPs was calculated using the following Equation (2).

Inhibition (%) = [(Abs Control − Abs Sample)/Abs Control] × 100(2)

### 3.9. Anti-Cancer

#### 3.9.1. Cell Culture

MCF-7 cells were cultured in DMEM with 10% FBS and 1% penicillin-streptomycin. MCF-7 cells were incubated at 37 °C in 5% CO_2_, and the medium was changed every two days.

#### 3.9.2. Cell Viability (%)

The cytotoxic activity of BM-AGNPs against MCF-7 was performed through an MTT assay. MCF-7 cells were cultured and 1 × 10^5^ cells/well were seeded into the wells and incubated overnight for 24 h at 37 °C in a 5% CO_2_ environment for cell attachment. The attached cells were treated with the BM extract and BM-AgNPs at various concentrations (1, 5, 10, 25, 50, 100, and 250 μg/mL) and cultured overnight for 24 h. The MTT solution dissolved in PBS was adjusted to 2 mg/mL, and then 50 μL was added to each well containing the cell and incubated for an additional 4 h. After inhaling the MTT solution, the formed purple formazan crystals were dissolved by adding 100 μL of DMSO. The absorbance was recorded at 540 nm using a microplate reader. Cell viability (%) was determined by substituting the absorbance value using the following Formula (3):

Cell viability (%) = (Abs of samples/Abs of controls) × 100(3)

### 3.10. Statistical Analysis

Analysis data were expressed as mean ± standard deviation using SAS version 9.4 (SAS Institute Inc., Cary, NC, USA) and were performed in triplicate. One-way analysis of variance (ANOVA) with Duncan’s multiple range test (DMRT) was used to compare significant differences between samples at the 5% level (*p* < 0.05).

## 4. Conclusions

In this study, biosynthesis of AgNPs was performed from AgNO_3_ using BM extracts serving as capping and reducing agents, and an environmentally friendly and cost-effective approach was adopted. The study results suggest the efficient application of green-synthesized nanoparticles in pharmaceuticals using BM extracts. The prepared AgNPs were characterized in structural and morphological aspects with antioxidant activity, anti-inflammatory, and dose-dependent cytotoxicity against MCF-7 breast cancer cells. UV-vis spectra confirmed the formation of AgNPs by showing an absorption peak for Ag nanoparticles at 437 nm. The crystalline structure of the biosynthesized AgNPs was confirmed through the XRD study. The particle size of AgNPs was determined to be less than 100 nm through TEM. The zeta potential value of −56.6 ± 0.56 mV obtained from DLS indicates the stability of AgNPs, and the particle size was measured to be 170.17 ± 12.65 nm. The diameter obtained via DLS can typically be higher than the diameter measured through TEM. The PDI was measured at 0.281 ± 0.07, indicating relative monodispersity. BM-AgNPs were all higher than the BM extract in TFC, TPC, and TAC. The relatively high content of active molecules can suggest a correlation with improved antioxidant and antibacterial activity. In potential antioxidant activity and antibacterial studies, AgNPs showed activity compared to the extract and could be used to treat bacteria-related diseases. In addition, BM-AgNPs exhibited suppression of protein denaturation in BSA, showing an anti-inflammatory effect comparable to the control group treated with declofenac sodium as the concentration increased. MTT analysis confirmed significant anticancer properties of BM-AgNPs against human breast cancer, exhibiting cytotoxic even at low concentrations. These findings suggest that BM-AgNPs possess potential therapeutic effects on antioxidant, antibacterial, anti-inflammatory, and anticancer properties, making them suitable candidates for nano-drug formulations in the pharmaceutical industry.

## Figures and Tables

**Table 1:** Particle size (nm), PDI and Zeta potential (mV) of BM-AgNPs.

	Particle Size (nm)	PDI (Index)	Zeta Potential (mV)
BM-AgNPs	170.17 ± 12.65	0.281 ± 0.07	−56.6 ± 0.56

**Table 2:** TPC, TFC, TAC, and IC_50_ values of antioxidant activities of BM and BM-AgNPs. Data are expressed as means ± standard deviation (n = 3). Different letters (a–c) in each samples (BM extract, BM-AgNPs, and Ascorbic acid) (*p* < 0.05) indicate significant differences.

Sample	TPC	TFC	TAC	ABTS	DPPH
	GAE mg/g	QE mg/g	C3G mg/L	IC_50_ μg/mL	IC_50_ μg/mL
BM extract	24.57 ± 0.17 ^a^	8.07 ± 0.04 ^a^	1.04 ± 0.24 ^a^	312.18 ± 15.34 ^a^	335.35 ± 39.33 ^a^
BM-AgNPs	148.42 ± 2.33 ^b^	12.80 ± 0.65 ^b^	1.63 ± 0.60 ^a^	250.53 ± 24.28 ^a^	134.90 ± 26.29 ^b^
Ascorbic acid	-	-	-	12.76 ± 0.54 ^c^	11.36 ± 0.27 ^c^

**Table 3:** MIC and MBC value of BM extract and BM-AgNPs.

	BM Extract		BM-AgNPs	
	MIC (μg/mL)	MBC (μg/mL)	MIC (μg/mL)	MBC (μg/mL)
*S. aureus*	-	-	600	1000
*E. coli*	-	-	600	1200

- (growth bacteria).

## Data Availability

All data generated for this study are contained within the article.
